# Remarks on the Influence of Mental Cultivation and Mental Excitement upon the Mind

**Published:** 1846-06

**Authors:** 


### PART II.—REVIEWS.

### ARTICLE V.

*Remarks on the Influence of Mental Cultivation and Mental Excitement upon the Mind.* By AMARIAH BRIGHAM, M. D., Superintendant and Physician to the State Lunatic Asylum, Utica, N. Y. Third Edition. Philadelphia: Lea & Blanchard. 1845. pp. 204. (From Brautigam & Keen, Chicago.)

In evidence of the meritorious character of this little volume, and to show that its value is duly appreciated, both at home and abroad, we need only mention the fact, that it has passed through three editions in this country, in as many years, and has been republished both in Glasgow and Edinburgh, under the supervision and with the recommendation of such men as Robt. Macnish, M.D., and Jas. Simpson Esq.

"The object of this work," says the author, "is to awaken public attention to the importance of making some modification in the method of educating children, which now prevails in this country. It is intended to show the necessity of giving more attention to the health and growth of the body, and less to the cultivation of the mind, especially in early life, than is now given; to teach that man, at every period of his existence, should be considered both as a spiritual and material being—as influenced both by physical and moral causes, and that therefore all plans for his improvement should be formed, not from a partial view of his nature; but from a knowledge of his moral, intellectual, and physical powers, and of their development."

The subject under consideration is, evidently one demanding the attention and close investigation of every individual, but it is especially the duty of the physician, whose province is to direct and advise others upon such points, to make himself thoroughly acquainted with the facts and arguments bearing upon this subject. In our estimation, every page of the work before us would be perused by our readers with satisfaction, pleasure and profit; we shall therefore quote from it such passages as seems to us most interesting and instructive, concious as we are, of the difficulty of making selections from pages so nearly faultless.

Our author commences by showing, in a very logical and conclusive manner, that the *brain* is the material organ by which the mental faculties are manifested. In his arguments upon this point we find the following remarks upon *insanity*, which are interesting in a medical point of view, and valuable from the fact that they are the conclusions of a physician, of correct observation and much experience with this disease.

"The phrase *derangement of mind*," says he, "conveys an erroneous idea; for such derangement is only a symptom of disease in the head, and is not the primary affection. It is true, that moral and mental causes may produce insanity, but they produce it by first occasioning either functional or organic disease of the brain. On examining the heads of those who die insane, some disease of the brain or its appendages is generally found. I am aware of the statement by many writers, that they have examined heads of the insane, and found no trace of organic disease. But, until late years, there has not usually been great accuracy in such examinations, and slight organic disease might have been overlooked. Even admitting that there was no organic disease in the cases described by these writers, there was undoubtedly functional disease inappreciable by the senses; just as there is often great disorder of the stomach and derangement of digestion which cannot be discovered by dissection. There are in fact no diseases which are independent of affected organs, although the affection may not always be evident to the senses.

"Although mental derangement may perhaps sometimes occur in individuals who after death exhibit no trace of organic disease, I think such cases are more rare than has generally been supposed. Dr. Haslam says, that insanity is always connected with organic alterations of the brain. Greding has noticed thickening of the skull in one hundred and sixty-seven cases out of two hundred and sixteen, besides other organic disease. Spurzheim says he *always* found changes of structure in the heads of insane people. M. Georget dissected a great number of brains, and his experience is conformable to that of the authors above-mentioned. Mr. Davidson, House Surgeon to the Lancaster County Lunatic Asylum, examined with great care the heads of two hundred patients who died in the asylum, 'and he scarcely met with a single instance in which traces of disease in the brain or its membranes were not evident, even when lunacy was recent, and a patient died of a different disease.'

"Dr. Wright, of the Bethlem Lunatic Hospital, states that in one hundred cases of insane individuals, whose heads he examined, all exhibited signs of disease; in *ninety* cases the signs were very distinct and palpable; in the remaining ten they were fainter, but still existed in some form or other,—such, for instance, as that of bloody points, when the brain was cut through.

"One of these writers for the prize offered some years ago, by the celebrated Esquirol, for the best dissertation on Insanity, observes, that he examined the heads of more than one hundred individuals who died from insanity, and comes to the following conclusions:—

"1st. That in the brains of those who die of insanity, changes of structure will always be found.

"2d. That these changes are the consequences of inflammation, either acute or chronic.

"3d. That there exists a correspondence between the symptoms and the organic changes; and that the names, monomania, mania, &c., ought only to be employed as representing degrees and stages of inflammation of the brain."

Numerous arguments and facts having been presented to prove most conclusively, that the brain is the organ for the manifestation of mind; this part of the subject is concluded as follows:

"I might adduce many more cases to prove the very intimate connexion between the brain and the mind, that it is a defective brain which makes the idot, and a diseased brain which causes delirium and insanity; and that all the various states of mind produced by alcohol or by opium, &c., arise from the disordered action which these articles produce in the brain; that the weak mind manifested by the infant, and the feeble mind by the aged, are produced by a small and undeveloped, or an enfeebled and disease brain, and not by a change of the immaterial mind itself. But cases enough have been cited to prove these truths. And if we do admit that the brain is the organ by which the mind acts, we must acknowledge the necessity of guarding this organ most carefully, of exercising it with extreme caution, of not endangering its delicate structure at any period of life by too much labor, or preventing its full development by too little; for the regular exercise of all the organs of the brain is necessary to prepare them for the active and powerful manifestation of the mental faculties.

"The healthy condition and proper exercise of the brain, are therefore far more important than of any other organ of the body, for we might as well expect good digestion with a diseased stomach, or good music from a broken instrument, as a good mind with a disordered, enfeebled, or improperly developed brain. And yet, how little regard has been paid to these important truths, in the cultivation of the mind!

While people are exceedingly fearful of enfeebling and destroying digestion, by exciting and overtasking the stomach, they do not appear to think they may enfeeble or derange the operation of the mind by exciting the brain, by tasking it when it is tender and imperfectly developed, as it is in childhood."

Our author next calls the attention of his readers, to the condition of the brain in early life, and to the effects of this condition upon the mind's manifestations.

"During childhood it" (the brain) "is 'very soft, and even almost liquid under the finger, and its different parts cannot be clearly distinguished.' Still at this time it is supplied with more blood, in proportion to its size than at any subsequent period. It then grows most rapidly, and more rapidly than any other organ: its weight is nearly doubled at the end of the first six months; and hence the nervous system, being connected with the brain, is early developed, and becomes the predominating system, in youth. At this period of life, however, which is devoted to the increase of the body, it is necessary that the nervous system should predominate; for this system is the source of all vital movement, and presides over, and gives energy to those actions which tend to the growth of the organization.—Besides, 'Infancy,' says Bichat, 'is the age of sensation. As everything is new to the infant, everything attracts its eyes, ears, nostrils, &c. That which to us is an object of indifference, is to it a source of pleasure. It was then necessary that the nervous cerebral system should be adapted by its early development to the degree of action which it is then to have.'

"But this great and early development, though necessary for the above purposes, very much increases the liability to disease; it gives a tendency to convulsions, and to inflammation and dropsy of the brain, and to other diseases of the nervous system, which are most common and fatal in childhood.

"It is, therefore, deeply important, that the natural action of the nervous system should not be much increased, either by too much exercise of the mind, or too strong excitement of the feelings, lest at the same time the liability of children to nervous diseases be increased, and such a predominance given to this system as to make it always easily excited, and disposed to sympathize with disorder in any part of the body; thus generating a predisposition to hypochondriasis and numerous afflicting nervous affections.

* * * "Dangerous forms of scrofulous disease among children, have repeatedly fallen under my observation, for which I could not account in any other way, than by supposing that the brain had been exercised, at the expense of other parts of the system, and at a time of life when nature is endeavouring to perfect all the organs of the body. And after the disease commenced, I have witnessed, with grief, the influence of the same cause, in retarding or preventing recovery. I have seen several affecting and melancholy instances of children, five or six years of age, lingering awhile with diseases, from which, those less gifted, readily recover; and at last dying, notwithstanding the utmost efforts to restore them. During their sickness, they constantly manifested a passion for books, and mental excitement, and were admired for the maturity of their minds. The chance for the recovery of such precocious children, is in my opinion, small, when attacked by disease; and several medical men have informed me that their own observations had led them to form the same opinion; and have remarked, that in two cases of sickness, if one of the patients was a child of superior and highly cultivated mental powers, and the other one equally sick, but whose mind has not been excited by study, they should feel much less confident of the recovery of the former than of the latter. This mental precocity, results from an unnatural development of one organ of the body, at the expense of the constitution, as is thus explained by two of the most celebrated men of the medical profession. 'It is a fundamental law of the distribution of vital powers,' says Bichat, 'that when they are increased in one part, they are diminished in all the rest of the living economy; that the sum is never augmented, but that they are necessarily transported from one organ to another, and therefore to increase the powers of one organ, it is absolutely necessary they should be diminished in the others.' 'Extra development and sensibility of the brain,' says Dr. James Johnson, 'cannot take place, but at the expense of some function or structure in the animal or organic system; when, therefore, an undue share of the vital energy of an individual is directed to a particular organ or system, a proportionate subduction is made from some other organ or system; and this is a most undoubted and most important truth, which is little understood, and less attended to by the world in general.'

* * * * "I would have the parent, therefore, understand that his child may be made to excel in almost any thing; that by increasing the power of certain organs through exercise, he can be made a prodigy of early mental or muscular activity. But I would have him at the same time, understand the conditions upon which this can be effected, and its consequences. I would have him fully aware, that in each case, unusual activity and power are produced by extraordinary development of an organ; and especially that in early life, no one organ of the body can be disproportionately exercised, without the risk of most injurious consequences. Either the over-excited and over-tasked organ itself will be injured for life, or the development of other and essential parts of the system will be arrested forever. From what has been said hitherto, we gather the following facts, which should be made the basis of all instruction; facts, which I often wish to repeat. *The brain is the material organ by which all the mental faculties are manifested; it is exceedingly delicate, and but partially developed in childhood; over-excitement of it when in this state, is extremely hazardous.*"

Upon the consequences which result from inattention to the connection between the mind and body, our author goes on to say that

"Teachers of youth, in general, appear to think, that in exciting the mind, they are exercising something totally independent of the body,—some mysterious entity, whose operations do not require any corporeal assistance. They endeavour to accelerate, to the utmost, the movements of an extremely delicate machine, while most unfortunately they are totally ignorant or regardless of its dependence on the body. They know that its action and power may both be increased for a while, by the application of a certain force; and when the action becomes deranged, and the power destroyed, they know not what is the difficulty, nor how it can be remedied. Fortunately they do not attempt to remedy it themselves, but call in the physician, who, if he affords any relief at all, does it by operating on a material organ. If medical men entertained the same views as teachers, they would, in attempting to restore a deranged mind, entirely overlook the agency of the body, and instead of using means calculated to effect a change of action in the brain, would rely solely upon arguments and appeals to the understanding. For if the mind may be cultivated independent of the body, why may not its disorders be removed without reference to the body?

* * * "The method of teaching little children varies in different schools; but that is everywhere considered the *best*, which forces the infant mind the *fastest*. In some schools the *memory* is chiefly cultivated, and children are taught innumerable facts. Here we see those who are scarcely able to talk, exhibited as wonderful children. They are declared to be deserving of the highest praise, and prophesied about as giving promise of great distinction in future, because they are able to tell us who was the oldest man, and many other equally useful and important facts. They are also able to tell us many truths in Astronomy, Geometry, Chemistry, &c. &c., of which the innocent beings know about as much as do parrots of the jargon they deliver. In other schools, teachers are opposed to such practice; and say that a child should learn nothing but what he understands; that the memory should not alone be cultivated; therefore, they teach children that Methuselah was not only the oldest man, and nine hundred and sixty-nine years of age, but that he was the son of Enoch, and the grandfather of Noah, and that a year means 365 days, and a day 24 hours; and all this they teach, in order, as they say, that a child may *fully understand* what he learns. Other teachers say, that it is very wrong to *compel* a child to learn—very wrong indeed; and that he should learn no more than he will cheerfully: but though they do not gain their purpose by exciting *fear*, they awaken other passions of the strongest kind in the child, by a system of *rewards* and of *praise*. Now of all these methods, if there is any preference, it should be given to the first; for that is the least objectionable which has the least tendency to develop the mind, and awaken the passions prematurely. They must all, however, be wrong, if they call into action an organ which is but partially formed; for they do not conform to the requirements of the laws of nature, and wait for organs to be developed, before they are tasked.

"I beseech parents, therefore, to pause before they attempt to make prodigies of their own children. Though they may not destroy them by the measures they adopt to effect this purpose, yet they will surely enfeeble their bodies, and greatly dispose them to nervous affections. Early mental excitement will serve only to bring forth beautiful, but premature flowers, which are destined soon to wither away, without producing fruit.

"Let parents not lament, because their children do not exhibit uncommon powers of mind in early life, or because, compared with some other children, they are deficient in knowledge derived from books. Let them rather rejoice if their children reach the age of six or seven, with well-formed bodies, good health, and no vicious tendencies, though they be at the same time ignorant of every letter of the alphabet. If they are in this condition, it is not to be inferred that their minds are inferior to those of children who have been constantly instructed. It is a great mistake to suppose that children acquire no knowledge while engaed in voluntary play and amusements.

"They thus do acquire knowledge as important as is ever acquired at school, and acquire it with equal rapidity. Many think that the child who has spent the day in constructing his little dam, and his mill, in the brook, or the stream that runs in the gutter; or in rearing his house of clods or of snow, or in making himself a sled or cart, has been but idle, and deserves censure for a waste of his time, and a failure to learn anything. But this is a great error of judgment; for, while he has thus followed the dictates of nature, both his mind and body have been active, and thereby improved. To him anything which he sees and hears and feels is new, and nature teaches him to examine the causes of his various sensations, and of the phenomena which he witnesses. For him, the Book of Nature is the *best book*, and if he is permitted to go forth among the wonders of creation, he will gather instruction by the eye, the ear, and by all his senses.

"He is for a while just as ignorant that stones are hard, that snow will melt, that ice is cold, that a fall from the tree will hurt him, and a thousand other common facts, as he is of a 'parallelogram,' or 'perimeter,' or 'the diameter of the sun,' or the 'pericarpium of flowers,' or of many other things, which some think important for infants to know. If his time is constantly occupied in learning the last, he will grow up ignorant of many commonn truths, and fail in the best of all learning, *common sense.*

"The remarks which I have made relative to the danger of too early exerting and developing the minds of children, are not made without some knowledge of the education of children in various parts of our country.

"That children *do* have their mental powers prematurely tasked, is a fact which I know, from personal observation. I have seen a course like the following pursued in many families in various parts of the country, and I know that this course is approved of by many excellent persons. Children of both sexes are required, or induced, to commit to memory many verses, texts of scripture, stories, &c., before they are three years of age. They commence attending school, for six hours each day, before the age of four, and often before the age of three; where they are instructed during three years in reading, geography, astronomy, history, arithmetic, geometry, chemistry, botany, natural history, &c. &c. They also commit to memory, while at school, many hymns, portions of the scriptures, catechisms, &c. During the same period, they attend every Sunday a Sabbath school, and there recite long lessons: some are required to attend upon divine service at the church twice each Sunday, and to give some account of the sermon. In addition to these labors, many children have numerous books, journals, or magazines to read, which are designed for youth. I have known some required to give strict attention to the chapter read in the family in the morning, and to give an account of it; and have been astonished and *alarmed* at the wonderful power of memory exhibited on such occasions by children when but five or six years of age. I have known other children, in addition to most of the above performances, induced to learn additional hymns, chapters of

Scripture, or to read certain books, by the promise of presents from their parents or friends.

"The foregoing account fails to describe the amount of mental labor required of many children in intelligent and respectable families.

"The injurious and sometimes fatal effects of such treatment have been already mentioned. But I cannot forbear again to state that I have myself seen many children who were supposed to possess almost miraculous mental powers, experiencing these effects and sinking under them. Some of them died early, when but six or eight years of age, but manifested, to the last, a maturity of understanding which only increased the agony of a separation. Their minds, like some of the fairest flowers, were 'no sooner blown than blasted.' Others have grown up to manhood, but with feeble bodies and a disordered nervous system which subjected them to hypochondriasis, dyspepsia, and all the Protean forms of nervous disease. Their minds, in some cases, remained active, but their earthly tenements were frail indeed. Others of the class of early prodigies, and I believe the most numerous portion, exhibit in manhood but small mental powers, and are the mere passive instruments of those who in early life were accounted far their inferiors. Of this fact I am assured, not only by the authority of books, and my own observation, but by the testimony of several celebrated teachers of youth."

With regard to the effect of inordinate mental cultivation and excitement in producing disease, our author remarks as follows:

"Intellectual cultivation, and powerful mental excitement, have a very important bearing upon one of the most appalling and deplorable diseases which afflicts humanity; a disease which now prevails to a great extent in this country, and is, I apprehend, increasing with fearful rapidity. The disease I allude to is *insanity*, or disorder of the organ of the mind, which produces a derangement in the manifestation of the mental faculties.

"We have no means of determining, correctly, the number of insane persons in the United States; but if there are as many in the other states of the Union as in Connecticut, the whole number cannot be less than *fifty thousand*, or *one in every two hundred and sixty-two* of the population.

"In Scotland, the proportion of insane to the population, is 1 to 574; and in the Agricultural districts of England, 1 to 820. There is, however, more insanity in England than in any other country of Europe.

"An inquiry, therefore, into the *causes* of so much insanity in this country becomes very important; and these causes must be sought among the agents that act upon the brain. I have already shown than insanity is a disease of the brain, and that whatever powerfully excites this organ, may so derange its action as to produce derangement of the mind. Sometimes it is occasioned by a *blow* or *fall* upon the head, at other times by inflammation or fever, which produces an unusual determination of blood to the brain. But far oftner the disease is occasioned by *moral causes*, by too violent excitement of the mind, producing morbid action in some parts of the brain.

"Thus we find that insanity prevails most in those countries where people enjoy civil and religious freedom, where every person has liberty to engage in the strife for the highest honors and stations in society, and where the road to wealth and distinction of every kind is equally open to all. There is but little insanity in those countries where the government is despotic. The inhabitants of such countries possess but little mental activity compared with those who live in a republic, or under a representative government.

* * * "In all ages and countries insanity has prevailed most in times of great moral and mental commotion. The crusades, and the spirit of chivalry that followed them, the reformation of Luther, the civil and religious discords of Europe, the French Revolution, the American Revolution, greatly multiplied cases of insanity. So true it is, that moral and mental causes excite this disease, that Esquirol says, he 'could give the history of the Revolution, from the taking of the Bastile until the last appearance of Bonaparte, by that of some lunatics, whose insanity relates to the events which have distinguished this long period.'

"Not only do the commotions which powerfully affect the minds of people occasion immediate insanity in adults, but they *predispose the next generation to this terrible disease;* and this is a fact that deserves great consideration. Esquirol says that many women, strongly affected by the events of the Revolution, bore children, whom the *slightest cause rendered insane.* He is supported by others in this opinion, *that strong mental emotion of the mother predisposes the offspring to insanity.*

* * * "In view of these few brief facts respecting *Insanity*, we are forced to believe, that among the causes of the great prevalence of this disease in this country, are the following:—

"1st. Too constant and too powerful excitement of the mind, which the strife for wealth, office, political distinction, and party success produces in this free country.

"2d. The predominance given to the nervous system, by too early cultivating the mind and exciting the feelings of children.

"3d. Neglect of Physical education, or the equal and proper development of all the organs of the body.

"4th. The general and powerful excitement of the female mind. Little attention is given in the education of females, to the physiological differences of the sexes. Teachers seldom reflect, that in them the nervous system naturally predominates; that they are endowed with quicker sensibility, and far more active imagination, than men; that their emotions are more intense, and their senses alive to more delicate impressions; and they therefore require great attention, lest their exquisite sensibility, which, when properly and naturally developed, constitutes the greatest excellence of women, should either become *excessive* by too strong excitement, or suppressed by misdirected education. If here was the proper place, it would be easy to show that efforts to make females excel in certain qualities of mind which in men is considered most desirable,—to make them as capable as men, of long continued attention to abstract truths, would be to act contrary to the dictates of nature, as manifested in their organization, and would tend to suppress all those finer sensibilities, which render them, in everything that relates to sentiment and affection, far superior to men.

"But in general the mental peculiarities of the female mind are not regarded in education. Their intellectual powers are developed to the greatest degree, and thus their natural sensibility is changed or rendered excessive. This excessive sensibility is not always counteracted by bodily labor or exercise; or there is probably no country where women belonging to the wealthy class, exercise so little, especially in the open air, as in this. But they here participate more, perhaps, than in any other country, in the excitement of parties and sects, which, in beings whose nervous system is easily excited, is very likely to produce strong emotions; and, as I have shown, such emotions may have deplorable effects upon their offspring.

* * * "There is another, and I fear a more frequent and fatal disease than that of insanity, caused by mental excitement; and which, judging from my own observation, and the records of cases in modern medical journals, appears to be increasing with frightful rapidity. I allude to organic diseases of the heart. The heart is a vital organ, and its sound state is essential to the possession of good health. When we reflect, therefore, upon the powerful influence which the feelings have upon this organ, the change from its natural action, caused by anger, fear, love, joy, avarice, ambition, envy, revenge, and all those passions and feelings that agitate civilized society, we shall not wonder that the diseases of the heart have increased in modern times. This disease has also increased in all countries during times of great political and moral commotion. Corvisart says, 'it was more frequent in the horrible times of the French Revolution than in the usual calm of social life.'

"Testa, in a late work on diseases of the heart, states the same fact as regards agitated Italy. This author considers the powerful and irregular operation of the passions, as the most frequent cause of organic disease of the heart. Whoever reflects upon these facts, must feel the importance of cultivating a quiet state of the mind in order to preserve good health. This is important at all times of life, but particularly so during childhood. It should be recollected that the early development of the mental powers of children awakens the passions and appetites earlier than they would be but for this premature mental cultivation, and therefore excites the heart while it is in a tender and delicate state."

Want of space compels us to pass over that portion of the work in which the author treats of excessive mental cultivation and cerebral irritation, as a cause of dyspepsia. His views upon this disease are peculiar, and his facts and arguments in support of them strong, if not conclusive.

We shall, therefore, as it is probable, in a succeeding number of the Journal, publish, in full, his remarks upon this subject. W. B. H.

